# Granulomatosis with polyangiitis: Common and uncommon presentations

**DOI:** 10.1111/1754-9485.13471

**Published:** 2022-09-20

**Authors:** Qiao Xin Tee, Aaron Wong, Mithun Nambiar, Kenneth K Lau

**Affiliations:** ^1^ Monash Imaging Monash Health Clayton Victoria Australia; ^2^ Faculty of Medicine, Nursing and Health Sciences Monash University Melbourne Victoria Australia; ^3^ Sir Peter MacCallum Department of Oncology University of Melbourne Melbourne Victoria Australia

**Keywords:** body CT, chest imaging, head and neck, magnetic resonance imaging, respiratory

## Abstract

Granulomatosis with polyangiitis (GPA) is a multisystemic autoimmune small vessel vasculitis predominantly affecting the respiratory and renal systems. Other systems such as the central nervous system, orbital, cardiac and gastrointestinal systems may also be involved to a lesser degree. Although there are no imaging features that are pathognomonic for GPA, there are known radiological patterns suggestive of the disease and imaging plays an important role in diagnosis, assessment and monitoring of disease activity. This is more evident when combined with clinical features, biochemical values and histopathology results. This pictorial review aims to present both common and uncommon radiological features of GPA.

## Introduction

Granulomatosis with polyangiitis (GPA), formerly known as Wegener's granulomatosis, is an uncommon multisystemic autoimmune disorder affecting small‐to‐medium‐sized vessels, characterised by pauci‐immune vasculitis and necrotising granulomatous inflammation most commonly involving the upper and lower respiratory tract and kidneys.^1^, ^2^ In this article, we present examples of both common and uncommon imaging findings of GPA above the diaphragm, as imaging manifestation of infra‐diaphragmatic pathology is rarely encountered.

### Clinical presentation

GPA can involve any organ system in a granulomatous or vasculitic pattern (Table 1). It often presents with upper respiratory symptoms such as rhinorrhoea, sinusitis, nasal obstruction and oral ulcers in 70–100% of patients with over 90% of patients developing lower respiratory tract involvement at some point during the course of their illness, experiencing symptoms such as haemoptysis, cough, dyspnoea and pleurisy.^3^, ^4^ Additionally, 83% of patients also experience renal involvement, presenting with a nephritic‐type syndrome with evidence of red cell casts.^3^ Whilst uncommon, orbital, central nervous system (CNS), skin, cardiac and gastrointestinal symptoms can occur.

**Table 1:** Summary of clinical and radiological features of GPA

Pathology	
Primary collagen necrosis Necrotising or palisading granulomas Micro‐abscesses with mixed cellular infiltrates including CD4+ T‐cells and macrophages Necrotising inflammation in blood vessel walls All of which can lead to thrombotic occlusion, rupture and haemorrhage	
**Clinical features**	**Radiological features**
*Pulmonary*	
Haemoptysis Cough Dyspnoea Pleurisy Chest pain	Pulmonary nodules with or without cavitation Feeding vessel sign Halo sign Reverse halo or Atoll sign Peripheral consolidation Reticulonodular ground‐glass infiltrates Haemorrhagic consolidation Tree‐in‐bud pattern or mosaic attenuation Wax and wane migratory pattern Pleural effusion Pleural granulomatous inflammation Fibrinous pleuritis Pleural thickening or nodularity Pneumothorax Circumferential trachea‐bronchial wall thickening or stenosis Increased broncho‐vascular lines Radiating linear scarring Pulmonary vessel irregularity Hilar lymphadenopathy
*Upper and middle airway (including nose and sinuses)*	
Oral ulcers Rhinorrhoea Chronic rhinosinusitis Epistaxis Mastoiditis Otitis Nasal polyps Nasal obstruction Saddle nose deformity Subglottic stenosis	Nasal turbinate hypertrophy Nasal septal erosion Tracheal thickening Intra‐ or extraluminal soft tissue thickening, masses, or granulomatosis Mucosal thickening Air‐fluid levels Bony or cartilaginous erosions or perforation Sinus tract formation Superimposed osteomyelitis
*Orbital*	
Conjunctivitis Episcleritis Scleritis Uveitis Retinitis Optic neuritis	Orbital inflammatory space occupying lesions Diffuse extraocular muscle swelling Orbital soft tissue masses Bony destruction in the orbital walls
*Cardiac*	
Non‐infectious endocarditis Heart failure Conduction abnormalities Valvular dysfunction Myocardial ischaemia from vasculitic occlusion	Pericarditis Myocarditis Coronary arteritis or thromboembolism
*Central Nervous System*	
Confusion Facial droop Limb weakness Ataxia Headache	Intracranial haemorrhage Cerebral ischaemia from vasculitic occlusion Intracranial or extracranial granulomatous lesions Cerebral and/or spinal pachymeningitis
*Gastrointestinal*	
Abdominal pain Diarrhoea Bloating Mucus or blood in stools Ascites Jaundice	Chronic inflammation of small bowel Pancreatic mass Acute pancreatitis Primary Biliary Cholangitis Autoimmune liver disease Primary Sclerosing Cholangitis
*Renal*	
Nephritic syndrome: Haematuria Peripheral oedema Decreased urine output Elevated blood pressure	Necrotising granulomatous pseudotumour lesions

The epidemiology of GPA within Australia is not well characterised but is likely similar to the estimated prevalence in the United States of around 3 per 100,000.^5^ It affects males and females equally and is more prevalent in Caucasians.^5^ The mean age of diagnosis is 40 years.^6^

GPA can be classified into systemic and limited disease, where ‘limited’ denotes disease confined to the respiratory tract without signs of systemic vasculitis.^7^ A firm diagnosis is usually made by histopathology of pulmonary or renal tissue showing necrotising granulomatous inflammation of small vessel walls.

The cause of GPA is unknown, but the presence of an autoimmune biomarker antineutrophil cytoplasmic antibody (ANCA) suggests that there is an autoimmune factor. ANCA is a group of antibodies targeting neutrophil constituents, activated through cross‐reaction with microbial antigens causing release of endothelial‐damaging reactive oxygen radicals and proteolytic enzymes, leading to necrotising inflammation in the blood vessel walls.^8^ Anti‐proteinase 3 (PR3‐ANCA), also known as c‐ANCA, specifically targets azurophilic granules in neutrophils and is associated with GPA. Accompanying the vasculitis and necrosis are granulomas, which may coalesce to form nodules that may cavitate. The process of granuloma formation is still unclear but is thought to be related to T‐cell hyperactivity with immunohistochemical studies confirming the presence of mainly CD4+ T‐cells and macrophages within cellular infiltrates in lung and kidney biopsies.^8^, ^9^

Although GPA does not have any reliable imaging features that are specific or pathognomonic for the disease, known radiological patterns suggestive of GPA exist and the choice of imaging depends on patient demographics and medical history, the body part or organ being examined and the clinical question being asked.

## Imaging findings

### Pulmonary manifestations

The radiographic appearance of GPA on chest imaging is variable, with 85% of GPA patients demonstrating abnormal findings on chest imaging, including pulmonary nodules, peripheral consolidation, reticulonodular ground‐glass infiltrates and pleural disease.^10^, ^11^

**Fig. 1:**
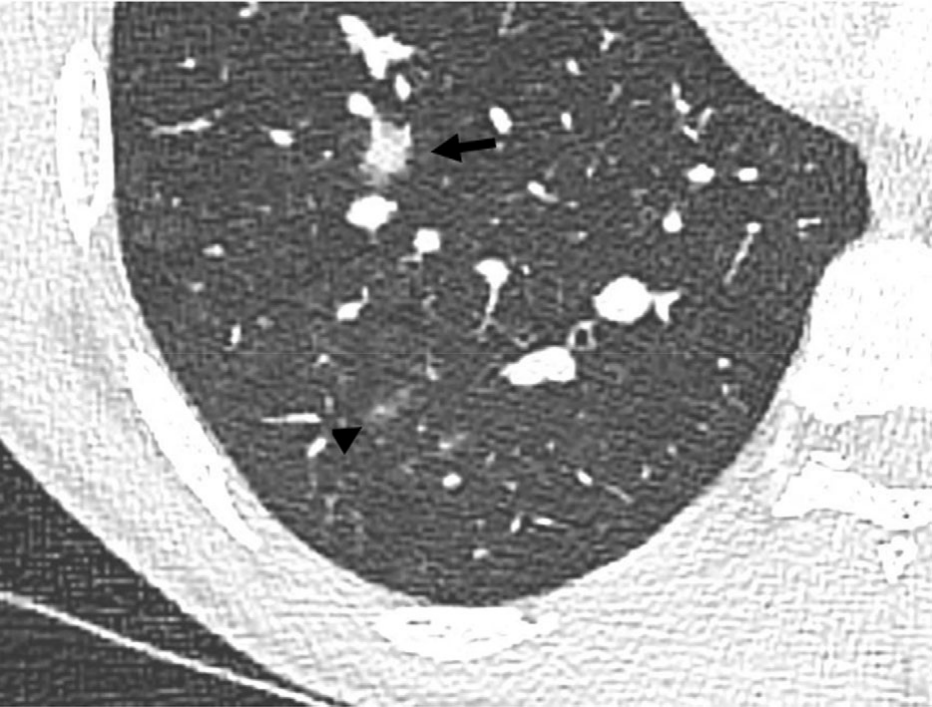
High resolution CT chest in a 24‐year‐old male patient diagnosed with GPA showing both irregular and ill‐defined centrilobular solid (arrow) and ground‐glass nodules (arrowhead), compatible with vasculitis.

**Fig. 2:**
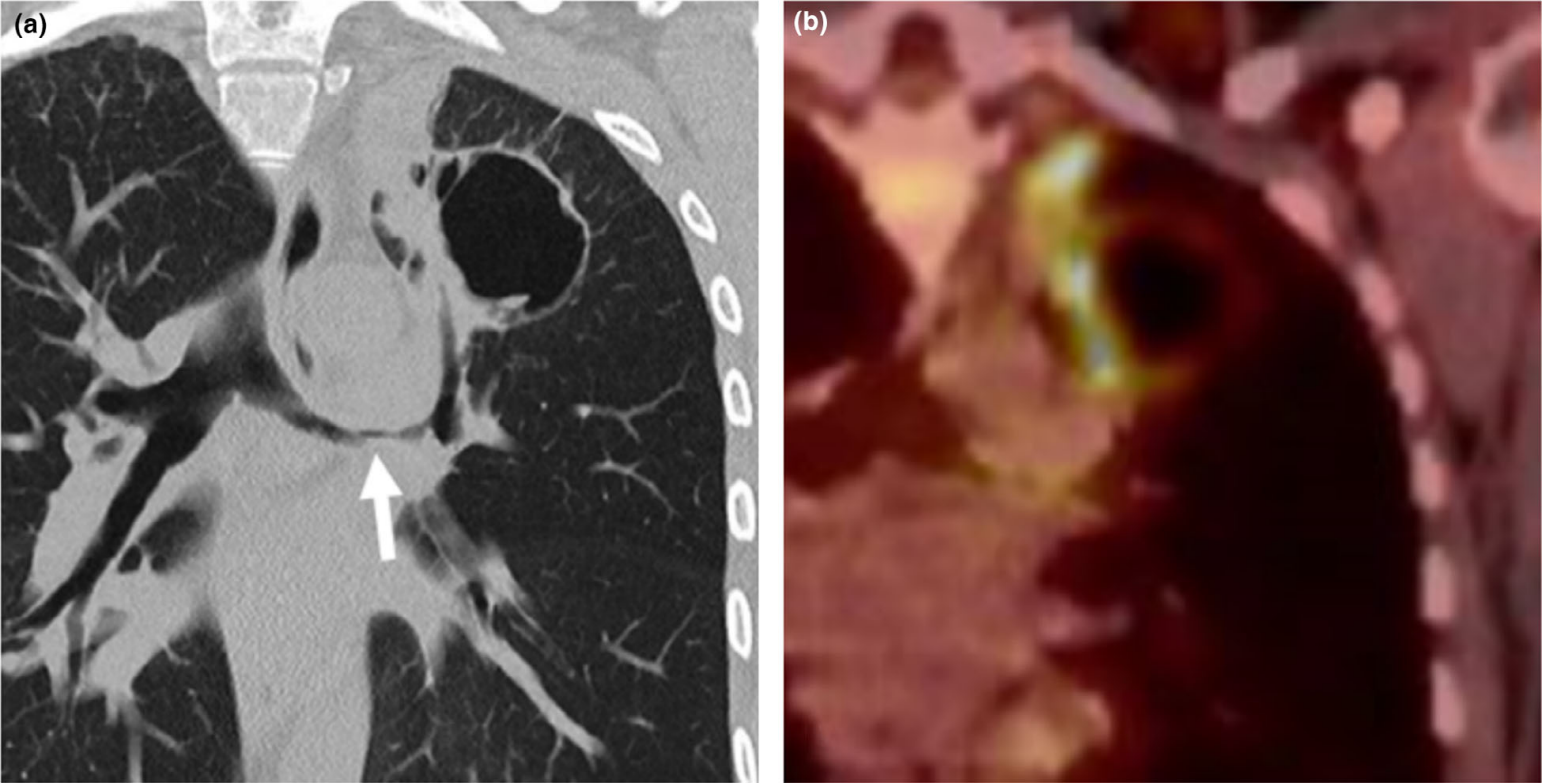
Coronal (a) reformats of a non‐contrast CT of a 34‐year‐old female demonstrating a lobulated cavitating nodule in the left upper lobe with irregular nodular wall thickening as well as marked irregular narrowing of the left main bronchus (white arrow). 18F‐Fluorodeoxyglucose (FDG) PET with a corresponding coronal (b) image showed avidity in its wall and septum, more marked along its medially thickened wall indicating this lesion was metabolically active. Subsequent resection of this lesion confirmed this was a GPA lesion.

**Fig. 3:**
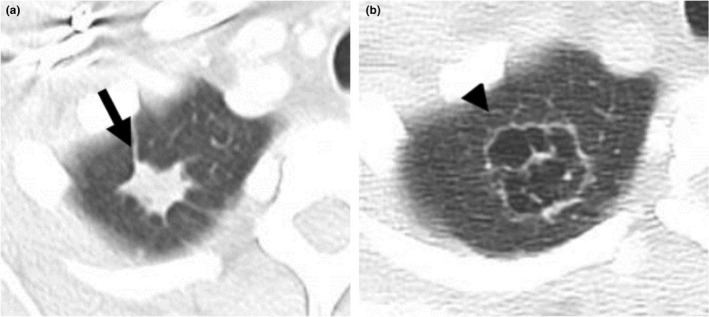
A 34‐year‐old female with a history of GPA was found to have an irregular nodule (black arrow) in the right lung apex on CT (a). This lung nodule showed cavitation (arrowhead) on the follow up CT 3 months later (b), which is in keeping with the evolution of GPA.

Pulmonary nodules are often multiple and bilateral with variable sizes ranging from a few millimetres up to 10 cm, with a broncho‐vascular or subpleural distribution.^12^ They often have irregular and ill‐defined margins (Fig. 1).^12^ Occasionally, the nodules are accompanied by a ‘feeding vessel sign’, where vessels are seen leading to the nodules, implying an angiocentric distribution.^12^ Nodules can also have a centrilobular distribution, mimicking other respiratory diseases such as acute pneumonia, hypersensitivity pneumonitis or tuberculosis.^6^ They are often heterogenous with variable densities and are seen in about 70% of patients, with half of the nodules showing cavities (Figs 2, 3). These can mimic metastasis or infection, including tuberculosis, abscesses and septic infarcts. In up to 75% of patients, biopsy would reveal primary collagen necrosis leading to characteristic pathognomonic necrotising or palisading granulomas and micro‐abscesses with mixed cellular infiltrates including mainly CD4+ T‐cells and macrophages as well as neutrophils, plasma cells and histiocytes.^11^, ^13^ Untreated nodules may grow or cavitate further, whilst treated nodules may resolve or develop into discoid scars.^14^

**Fig. 4:**
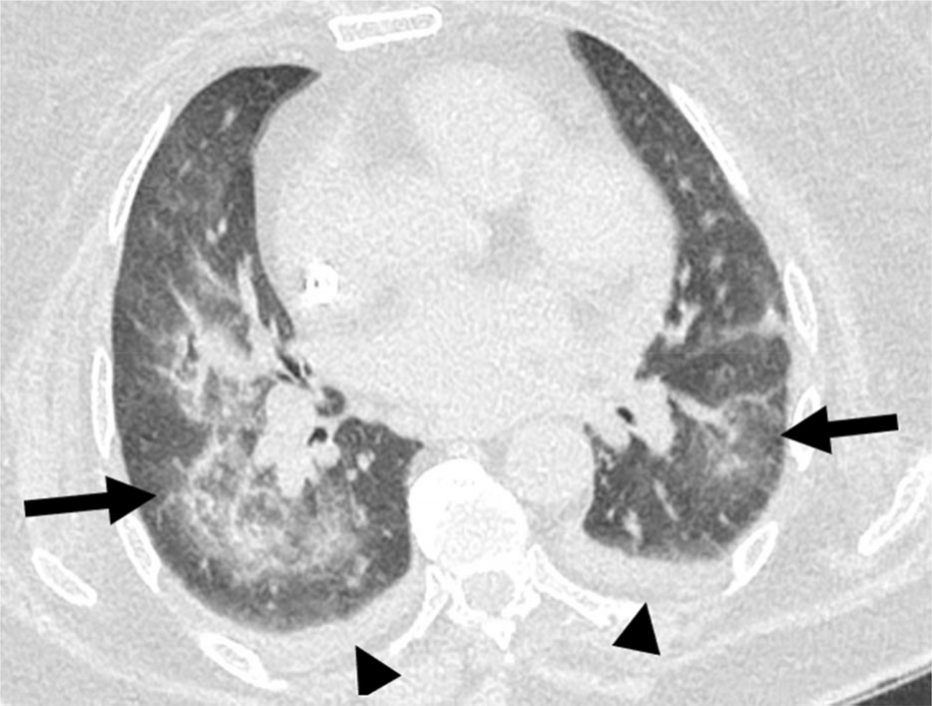
Non‐contrast axial CT chest of a 71‐year‐old female patient with End‐Stage Renal Failure secondary to GPA showing ground‐glass and air space densities throughout both lungs (arrows), which may represent diffuse alveolar haemorrhage in the setting of vasculitis. Small bilateral pleural effusions are also present (arrowheads).

Ground‐glass opacification or alveolar consolidation is seen in around 50% of patients, often affecting the mid‐to‐lower zones of the lung, in contrast to sarcoidosis, which predominantly affects the upper and middle zones of the lung. The ground‐glass or alveolar densities can be focal, subpleural, wedge‐shaped or cavitating, but quite commonly, have perihilar and peribronchovascular distributions.^13^ The pattern of consolidation is often dense and often contains air bronchograms. These features are often related to associated infarction, haemorrhage, intra‐alveolar cellular debris and/or granulomatous changes within the lung which are caused by necrotising inflamed capillaries, infiltrating inflammatory cells and formation of micro‐abscesses, leading to thrombotic occlusion, rupture and haemorrhage.^13^ The degree or severity of pulmonary haemorrhage determines whether ground glass or consolidative changes are seen on imaging, with ground glass changes correlating with microhaemorrhages and consolidation correlating to larger pulmonary haemorrhages (Fig. 4). These may be formed from the coalescence of smaller areas of diffuse haemorrhage into denser areas of haemorrhagic consolidation, which can be life threatening if not treated. A ‘tree‐in‐bud’ pattern or mosaic attenuation can be another manifestation when there is pulmonary arteriolar involvement.^12^

**Fig. 5:**
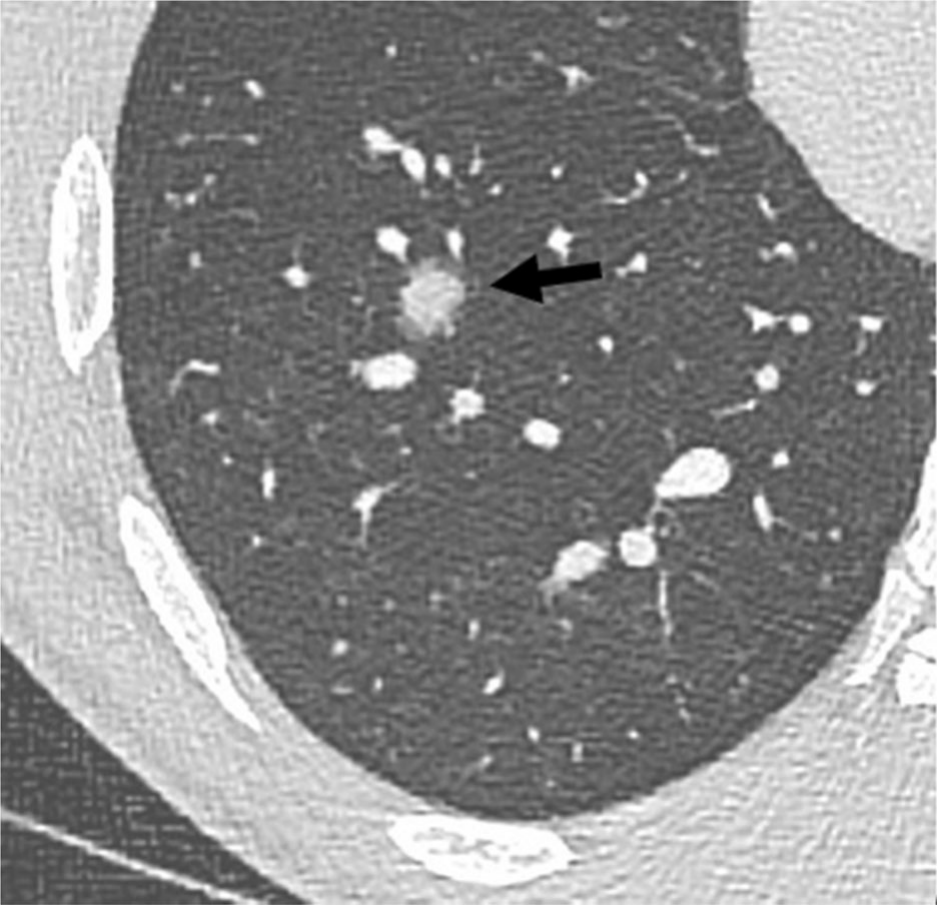
Post contrast axial CT chest with limited HRCT reformats of a 24‐year‐old male patient with GPA presenting with haemoptysis demonstrates a rim of ground‐glass opacity surrounding a consolidated nodule, known as the ‘halo’ sign (arrow).

**Fig. 6:**
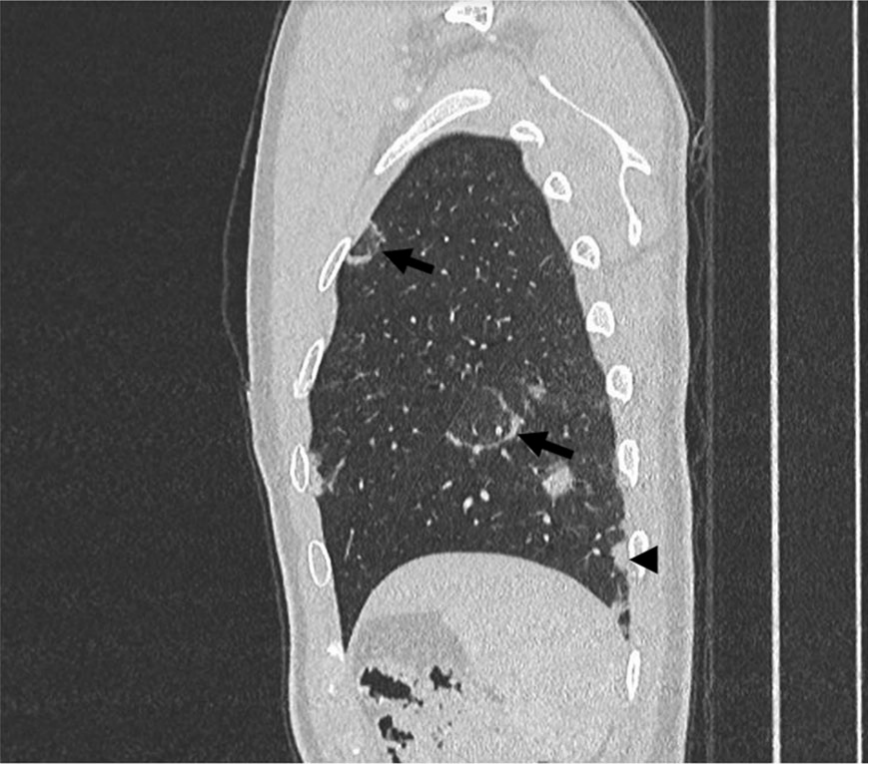
Sagittal CT chest of a 23‐year‐old patient with GPA showing multiple ill‐defined lung nodules affecting both upper and lower lobes, also with subpleural involvement (arrowhead). The Atoll sign is present as a ground‐glass nodule being surrounded by a thin rim of peripheral consolidation (arrows).

The halo sign, appearing as a rim of ground‐glass opacity surrounding a consolidated nodule, is often due to haemorrhage around a nodule affected by GPA and is present in around 15% of patients (Fig. 5).^6^ The reverse, known as the reverse halo or Atoll sign, is when there is an organising pneumonia reaction surrounding an area of focal haemorrhage, manifesting as a thin rim of peripheral consolidation surrounding a ground‐glass nodule (Fig. 6).^6^

**Fig. 7:**
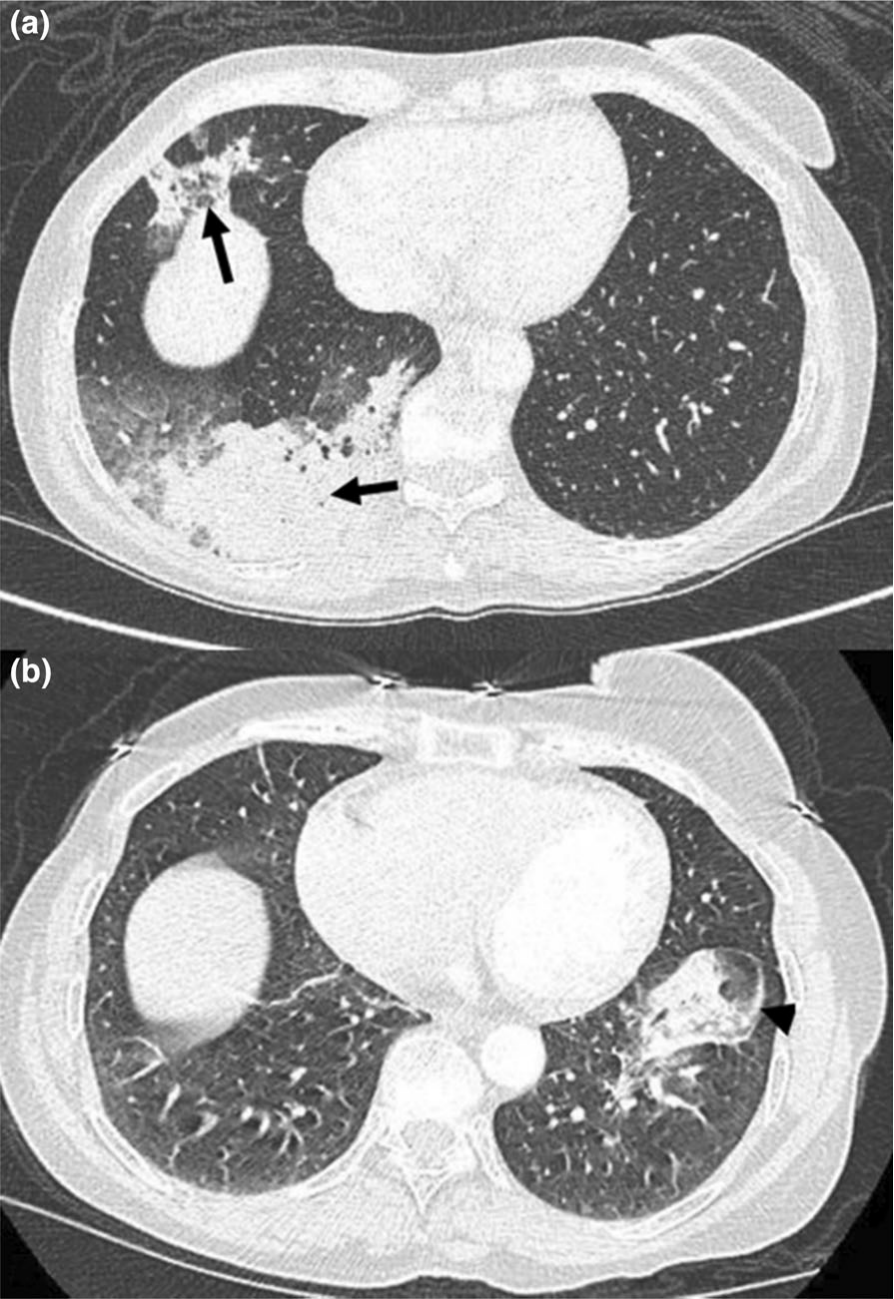
Axial CT chest in a 53‐year‐old female patient with GPA demonstrating confluent alveolar densities in the right middle and lower lobes (arrows) (a), which resolved 6 months later (b). However, new airspace density developed in the left lower lobe (arrowhead) (b), which would be consistent with a migratory pattern.

Pulmonary nodules, masses, ground glass or consolidative changes in GPA are best demonstrated on CT and often co‐exist in the same patient. They may wax or wane, exhibiting a migratory pattern regardless of the treatment phase (Fig. 7).^12^

Pleural involvement includes pleural effusion (seen in up to 20% of patients) (Fig. 4), pleural granulomatous inflammation (seen in up to 6% of patients), fibrinous pleuritis, pleural thickening, nodularity and pneumothorax (which are rare).^13^ Pleural effusions can be from primary pleural involvement or secondary to disease affecting the heart or kidneys.^15^, ^16^

Other findings include circumferential bronchial wall thickening (which can be smooth or nodular) (Fig. 2), stenosis, increased broncho‐vascular lines, pleural tags, radiating linear scarring and pulmonary vessel irregularity (best seen on pulmonary angiogram).^12^ Less common findings include mediastinal or hilar lymphadenopathy, interstitial disease and bronchiectasis.

### Upper and middle airway manifestations

**Fig. 8:**
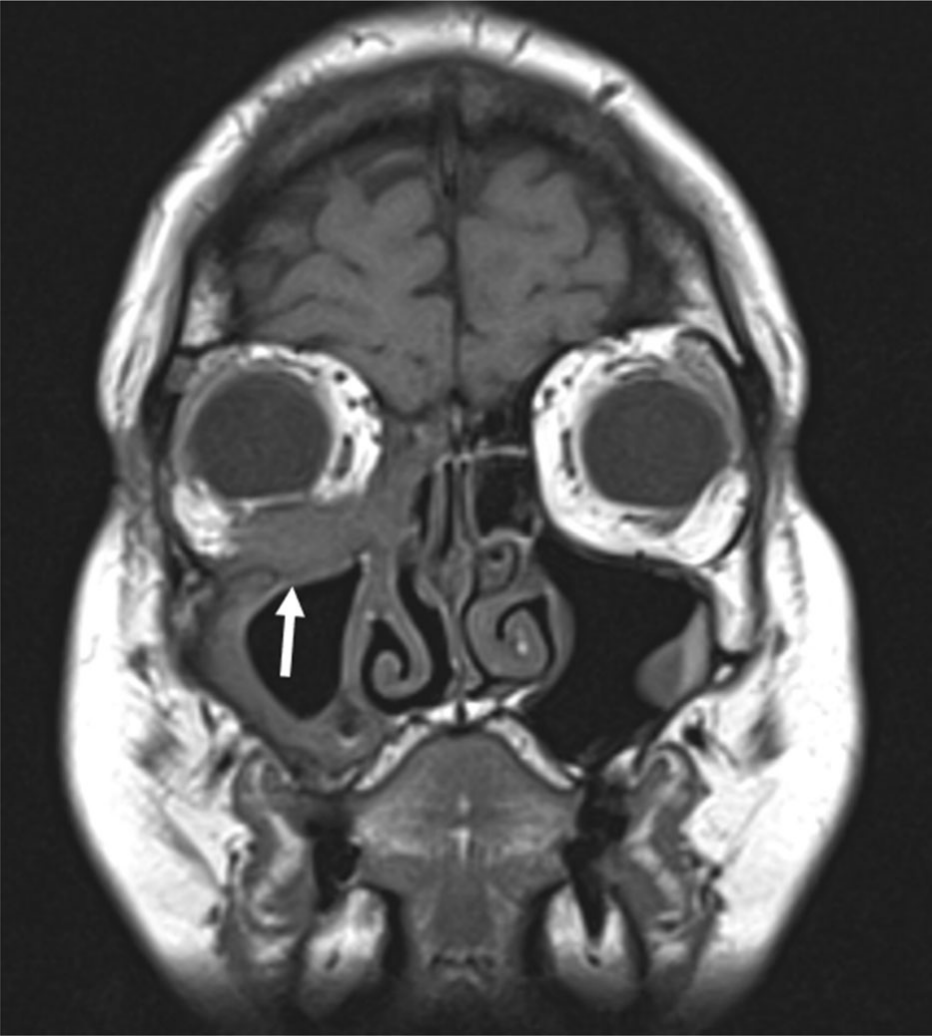
Coronal T1 weighted MRI orbit in a 49‐year‐old female patient with GPA showing a soft tissue mass with destruction of the floor of the right orbit extending into the right orbital inferior extraconal space that was inseparable from the adjacent inferior rectus (arrow). The patient was later diagnosed with GPA when biopsy of the right maxillary uncinate and ethmoid sinus showed necrotising granulomatous inflammation.

**Fig. 9:**
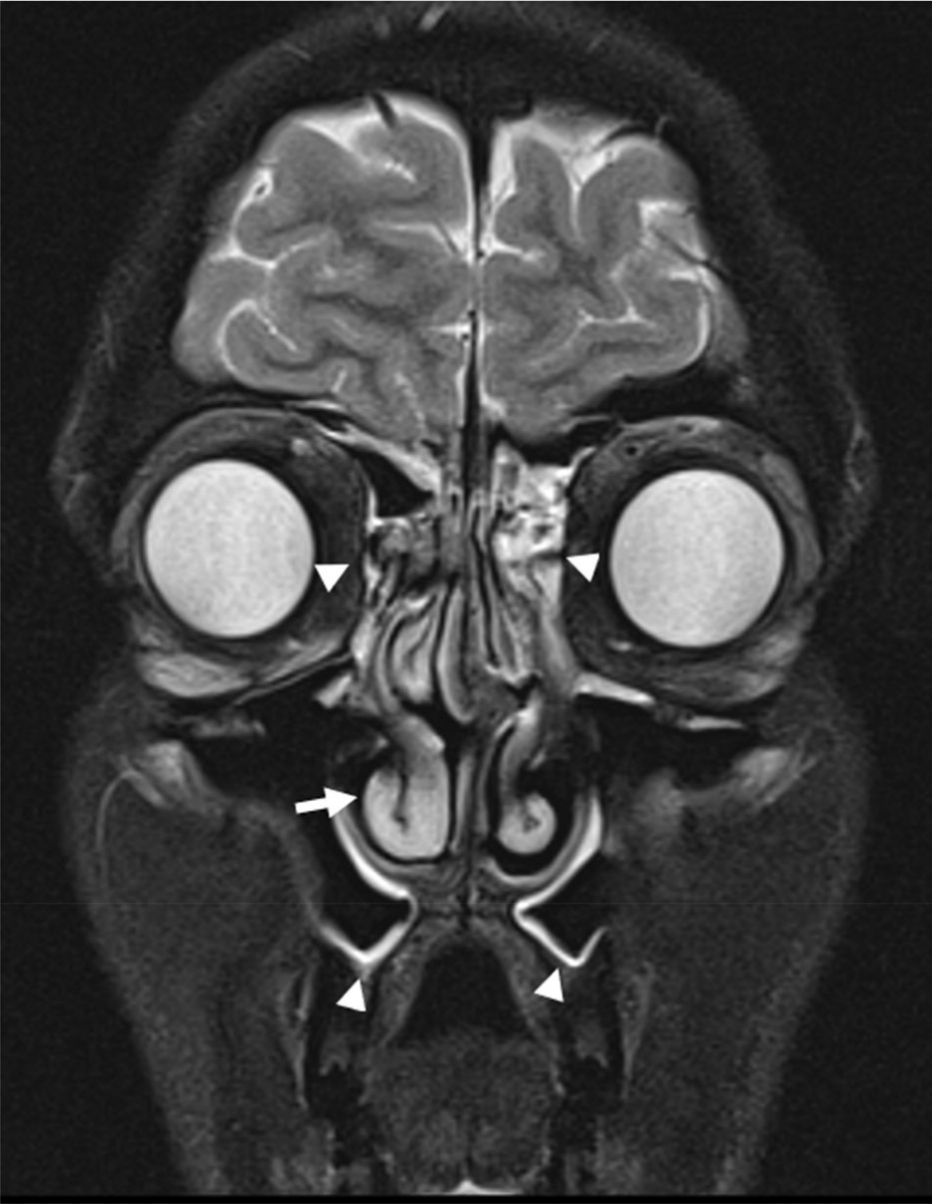
Coronal view of T2 weighted MRI brain and orbits in a 24‐year‐old male patient with GPA showing right inferior nasal turbinate hypertrophy (arrow) and mucosal thickening in the maxillary and ethmoid sinuses (arrowheads).

**Fig. 10:**
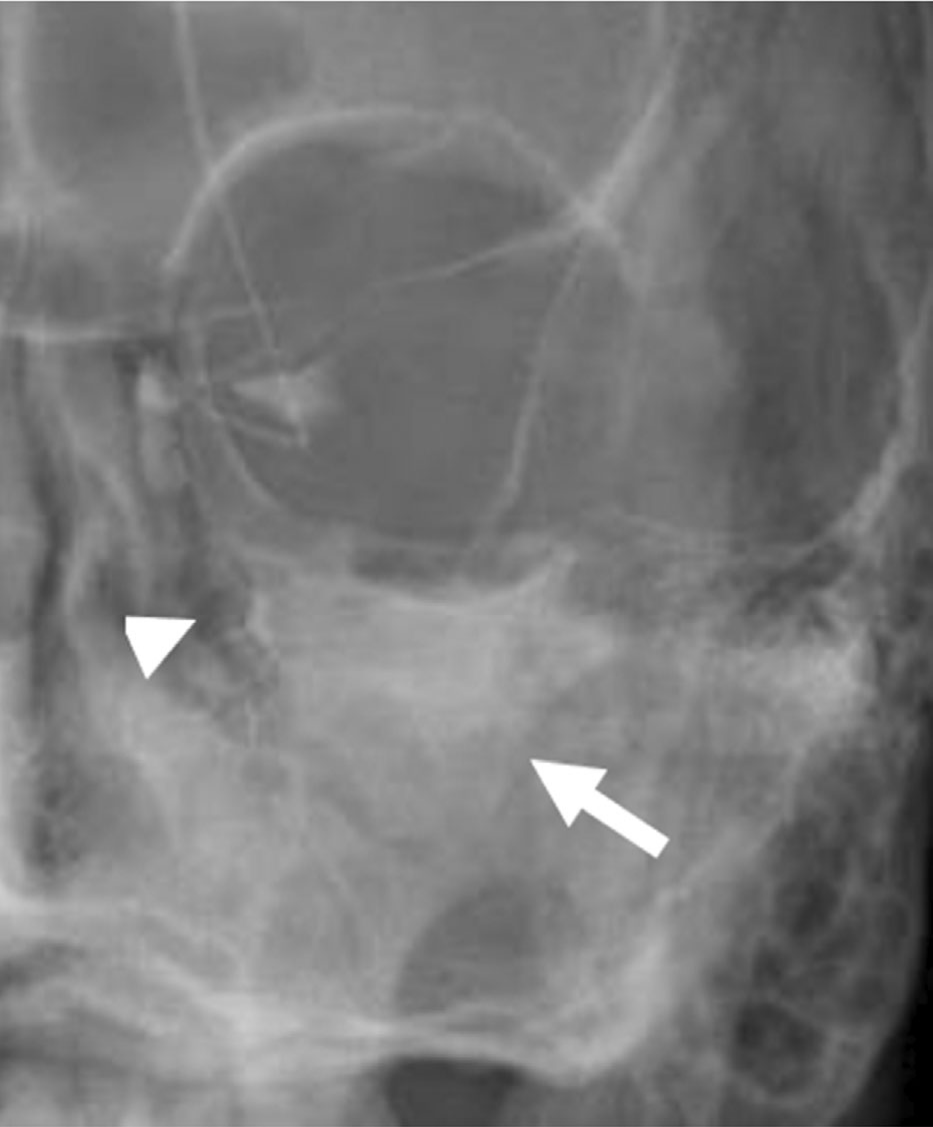
Left dacryocystogram of a 67‐year‐old male patient with GPA demonstrating a faint fistula (arrowhead) between the lacrimal duct and maxillary sinus, where contrast injected into the left lacrimal duct drained into the left maxillary sinus (arrow) instead of the left nasal cavity.

**Fig. 11:**
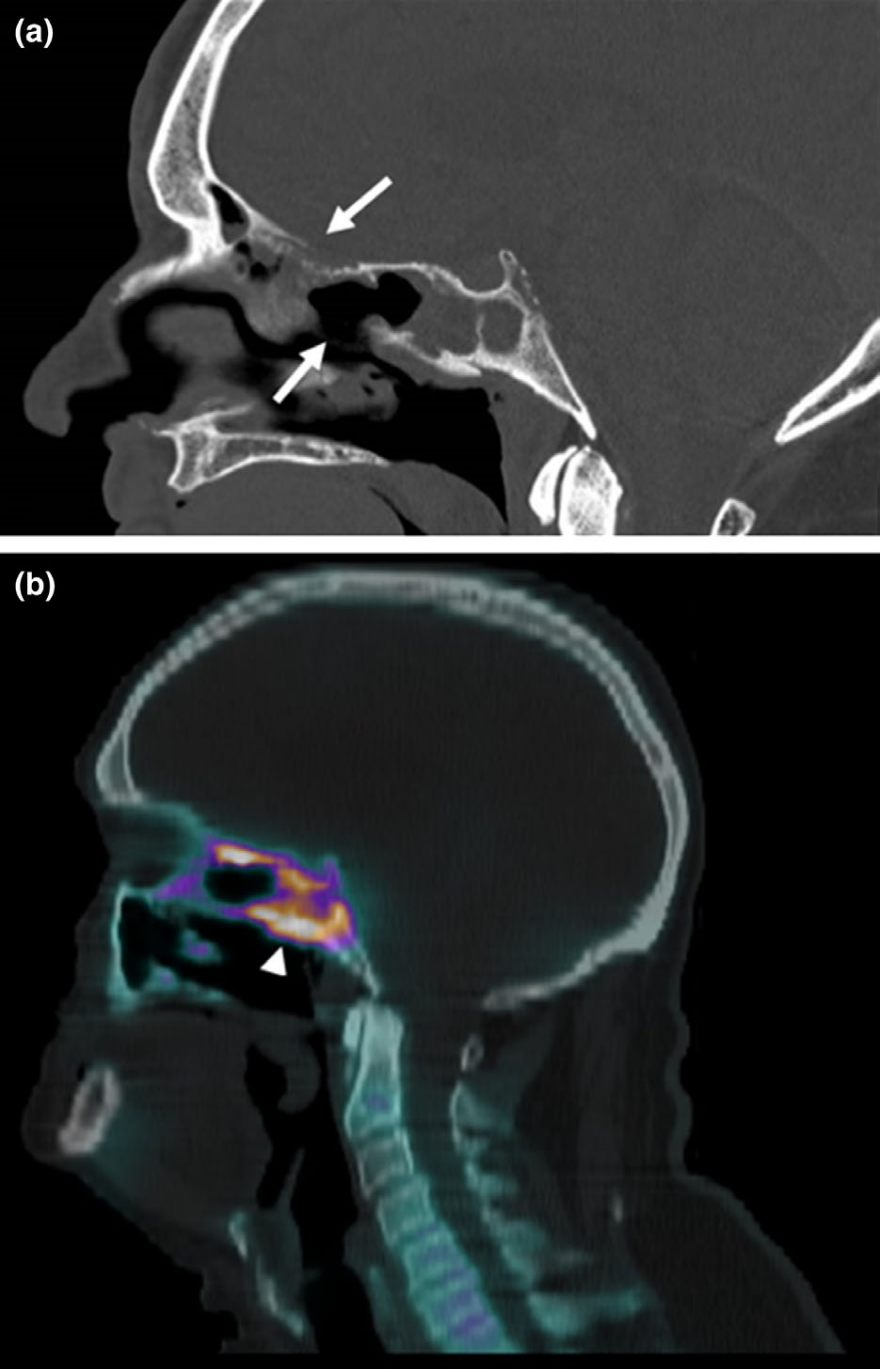
Non‐contrast sagittal reformat of CT paranasal sinuses (a) of a 72‐year‐old male patient with GPA showing bony destruction and osteomyelitis of the ethmoid and sphenoid bones (arrows). Focal hyperaemia and increased osteoblastic activity centred in the sphenoid bone extending to the sella turcica and clivus were present on the nuclear medicine bone scan (arrowhead) (b).

Although approximately half the GPA cases involve the airway, it is less often detected on imaging. Some upper respiratory features include chronic rhinosinusitis, epistaxis, mastoiditis, otitis, nasal polyps, nasal obstruction, nasal septal erosion, saddle nose deformity, intra‐ or extra‐luminal soft tissue thickening or masses of the larger airway, subglottic stenosis and tracheal calcification or thickening.^11^ Nasal saddle deformity is a result of nasal septum erosion and perforation caused by cartilage inflammation from frequent nosebleeds and nasal crusting. Soft tissue nodules, masses or granulomatosis (Fig. 8), mucosal thickening (Fig. 9), nasal turbinate hypertrophy (Fig. 9), air‐fluid levels and bony or cartilaginous erosions or perforation can affect any part of the upper respiratory tract including the nose and sinuses (Figs 8, 10, 11). In rare cases, a sinus tract between the lacrimal duct and maxillary sinus can be formed from the bony destruction as a result of vasculitis (Fig. 10). Increased uptake on nuclear medicine bone scan can be seen when there is superimposed infection with osteomyelitis of the associated sinus as a complication of chronic sinusitis (Fig. 11).

### Orbital manifestations

Ophthalmic manifestations of GPA are common and occur in up to 87% of patients, with 8–16% of patients presenting with orbital symptoms at diagnosis.^17^ This can include small vessel vasculitis involving any part of the eye such as conjunctivitis, episcleritis, scleritis, uveitis, retinitis and optic neuritis, most of which are often detected clinically rather than radiologically. Imaging findings include inflammatory space occupying lesions in the orbit, diffuse extraocular muscle swelling, orbital soft tissue masses and bone destruction in the orbital walls (Figs 8, 10), all of which may lead to compression of the optic nerve or proptosis.^18^ Other imaging findings include dacryocystitis and pre‐septal cellulitis.

### Cardiac manifestations

**Fig. 12:**
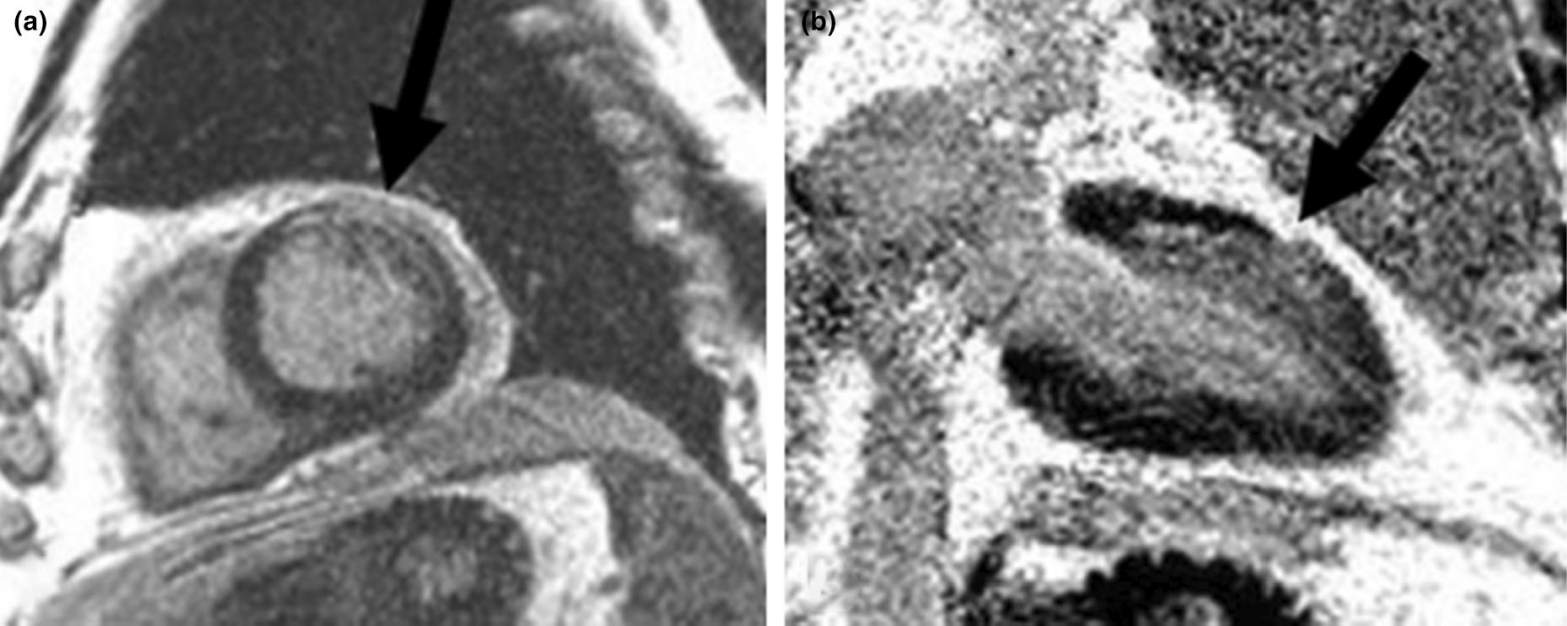
Sagittal (a) and coronal view (b) of a post gadolinium cardiac MRI of a 61‐year‐old male patient with GPA showing left ventricular wall myocardial late gadolinium enhancement in the anterior and anterolateral segments (arrows), indicative of myocarditis/fibrosis.

Literature reports a 6–25% prevalence of cardiac involvement in patients with GPA.^19^ Cardiac manifestations are associated with higher mortality and morbidity even though they are often not evident clinically. Inflammation can occur in any part of the heart, including pericarditis, myocarditis (Fig. 12), non‐infectious endocarditis, heart failure, conduction abnormalities, valvular dysfunction, coronary arteritis or thromboembolism and myocardial ischaemia resulting from vasculitic occlusion of coronary arteries.^19^

### Central nervous system manifestations

**Fig. 13:**
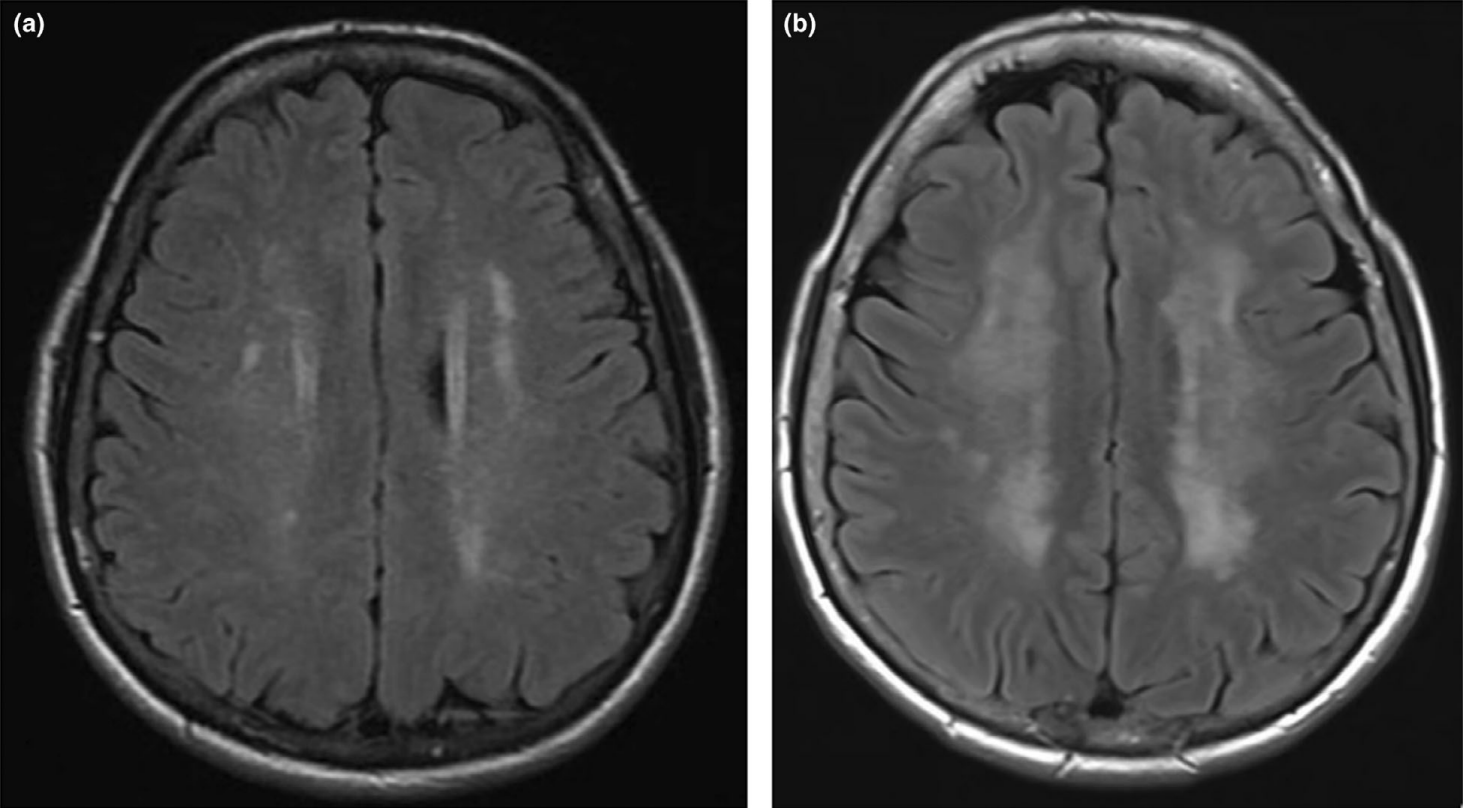
Axial FLAIR MRI brain in a 61‐year‐old female patient with GPA showing moderate amount of supratentorial deep white matter FLAIR hyperintensity in keeping with small vessel vasculopathy (a). This white matter ischaemia markedly progressed as compared to the previous MRI 5 years prior to this examination (b).

The central nervous system is affected in only 2–9% of patients with GPA. This can present as intracranial haemorrhage, ischaemia secondary to arterial occlusion from small vessel vasculitis involving the CNS (Fig. 13), intracranial or extracranial granulomatous lesions and cerebral and/or spinal pachymeningitis, which can be associated with long‐term neurological consequences.^20^

In conclusion, GPA is a multisystemic autoimmune small vessel vasculitis predominantly affecting the upper and lower respiratory tract, orbits and renal systems, and to a lesser extent, other systems. Although there are no reliable features on imaging that are pathognomonic for GPA, when combined with clinical features, biochemical values and histopathology results, imaging has an important role in making the diagnosis, assessing the extent of organ involvement, and monitoring the response to treatment.

## Data Availability

Data sharing is not applicable to this article as no new data were created or analyzed in this study.
